# Prevalence of Antibiotic Resistance of Haemophilus Influenzae in Iran- A Meta-Analysis

**DOI:** 10.22038/ijorl.2019.34363.2137

**Published:** 2019-11

**Authors:** Hamid Vaez, Amirhossein Sahebkar, Farhad Pourfarzi, Arshid Yousefi-Avarvand, Farzad Khademi

**Affiliations:** 1 Department of Microbiology, School of Medicine, Zabol University of Medical Sciences, Zabol, Iran.; 2 Biotechnology Research Center, Pharmaceutical Technology Institute, Mashhad University of Medical Sciences, Mashhad, Iran. Neurogenic Inflammation Research Center, Mashhad University of Medical Sciences, Mashhad, Iran.; 3 Department of Community Medicine, Ardabil University of Medical Science, Ardabil, Iran.; 4 Department of Microbiology, School of Medicine, Mashhad University of Medical Sciences, Mashhad, Iran.; 5 Department of Microbiology, School of Medicine, Ardabil University of Medical Sciences, Ardabil, Iran.

**Keywords:** Antibiotic resistance, H. influenzae, Iran

## Abstract

**Introduction:**

Based on the World Health Organization reports, infections caused by ampicillin-resistant Haemophilus influenzae strains are a major threat to public health and need urgent therapy with new antibiotics. Given the lack of a previous comprehensive study on the prevalence of the antibiotic resistance of H. influenzae in Iran, this systematic review and meta-analysis was performed to increase the knowledge about antibiotic resistance status of this pathogenic agent.

**Materials and Methods:**

For the purpose of the study, the articles related to the subject of interest and published up to August 2018 were searched in several English and Persian databases, including PubMed, Scopus, Web of Science, Scientific Information Database, and Magiran. The search process was accomplished using the following keywords: “Antibiotic resistance”, “H. influenzae”, and “Iran”. The data were pooled from 13 eligible studies reporting the prevalence of antibiotic resistance of H. influenzae in Iran.

**Results:**

The prevalence of H. influenzae resistance to various antibiotics in Iran, including ampicillin, amoxicillin, cephalexin, cefixime, ceftazidime, cefotaxime, and ceftizoxime, were obtained as 54.8%, 66.6%, 28.6%, 62%, 21.3%, 22.3%, 23.2%, respectively. These rates were reported as 27.7%, 46.7%, 53%, 82.6%, 40.3%, 30.8% for chloramphenicol, tetracycline, trimethoprim/sulfamethoxazole, penicillin, erythromycin, and ciprofloxacin, respectively. Additionally, ceftriaxone, gentamicin, amikacin, kanamycin, rifampin, azithromycin, and clindamycin had the H. influenzae resistance rates of 33.1%, 40.2%, 45.8%, 44.4%, 18.5%, 17.4%, and 71.3%, respectively.

**Conclusion:**

The majority of the antibiotics tested in Iran showed a high rate of resistance to H. influenzae. This may cause serious problems in the treatment of infections in the future. Therefore, precautionary measures, such as monitoring antibiotic prescription and resistance and using the new classes of antibiotics, are necessary.

## Introduction

Members of Haemophilus genus are small, non-motile, Gram-negative rods or coccobacilli (pleomorphic), and facultative anaerobic bacteria which are classified in the family Pasteurellaceae (1,2). Haemophilus influenzae is the most common Haemophilus species infecting human, as the only known reservoir, which exists asymptomatically in the naso- and oro-pharynx of healthy carriers (1-3). Its primary colonization occurs through binding to the mucous membranes of the upper respiratory tract where the bacterium interferes with the ciliary motion. Therefore, the major transmission route of this species is through the respiratory tract (1-5). 

Based on a polysaccharide capsule antigen, bacteria can be divided into encapsulated and noncapsulated/nontypeable (NTHi) strains. In addition, the encapsulated isolates can be categorized into six antigenic serotypes (a-f) (1-5). Encapsulated and nonencapsulated H. influenzae are responsible for several life-threatening invasive infections in children and adults. Some of these infections include acute bacterial meningitis, epiglottitis, pneumonia, bacteremia/sepsis, septic arthritis, otitis media, sinusitis, and cellulitis (1-5).

Haemophilus influenzae type b (Hib) is a common cause of severe diseases, almost exclusively among children under the age of 5 years. According to the World Health Organization (WHO) estimates performed in March 2012, Hib is responsible for 2% of all-cause child mortality and 199,000 deaths per year (6,7). A high morbidity rate in untreated patients, as well as serious neurologic sequelae, which is particularly due to H. influenzae meningitis or epiglottitis, calls for proper diagnosis and treatment with antimicrobial agents (1,2,5). The important antibiotics applied in the treatment of less severe H. influenzae infections include amoxicillin, cephalosporin, azithromycin, doxycycline, and fluoroquinolone. However, serious infections are managed by broad-spectrum cephalosporins and carbapenems (1,2). Additionally, rifampin is the drug of choice for antibiotic prophylaxis in children carrying Hib (2). Nonetheless, WHO has recently placed ampicillin-resistant H. influenzae strains, along with penicillin-resistant Streptococcus pneumoniae and fluoroquinolone-resistant Shigella strains, in the list of antibiotic-resistant bacteria and medium priority category, in terms of the emergency of developing new antibiotics (8). Therefore, assessing antimicrobial susceptibility patterns and monitoring the resistance trend of H. influenzae are essential to guide prior antibiotic choice and prescription at the local level, thereby reducing the risk of treatment failure. With this background in mind, the present study was conducted to review the evidence on the antimicrobial susceptibility patterns of H. influenzae strains to different antibiotics in Iran through a systematic review and meta-analysis.

## Materials and Methods

**Search strategies**

To find all reports on the prevalence of the antibiotic resistance of H. influenzae in Iran, the related studies published up to August 2018 were searched in several electronic databases, including PubMed, Scopus, Web of Science, Scientific Information Database (SID), and Magiran. “Antibiotic resistance”, “H. influenzae”, and “Iran” were the most important MeSH-extracted keywords. In addition, the manual search of the bibliographies was performed to avoid missing any relevant articles. This systematic review and meta-analysis was performed in accordance with the Preferred Reporting Items for Systematic reviews and Meta-Analyses (PRISMA) checklist (9).

*Selection of articles*

**Fig 1:**
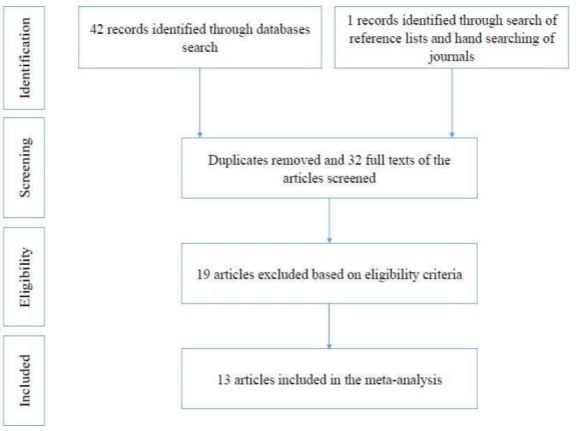
Schematic representation of the article selection process

Screening of the articles was performed in three steps by two independent authors based on the defined inclusion and exclusion criteria. The titles, abstracts, and full texts of the articles were sequentially reviewed (Fig.1). 

All kinds of articles that were published in English or Persian languages (with the exception of review articles and duplicates) and reported on the antibiotic resistance of H. influenzae in Iran were included in the study. According to the Newcastle-Ottawa quality assessment scale criteria adapted for cross-sectional studies, a quality assessment of the included studies was performed on the meta-analysis, and high-quality studies received 5 or more stars.

*Data extraction*

Data collection process from eligible studies was performed in duplicate (Table.1). The main extracted data from the included studies were the year of study, city, number of isolated H. influenzae strains, antibiotic susceptibility testing methods, and number of resistant strains to different antibiotics.

*Meta-analysis*

Quantitative data synthesis was performed using Comprehensive Meta-Analysis software (version 2.2; Biostat, Englewood, NJ). The rate of H. influenzae antimicrobial resistance was reported as percentage and 95% confidence intervals (95% CIs) using fixed- or the random-effects models. In case of observing a high heterogeneity (I^2^ statistic>25% and P<0.1) among the included studies, a random-effects model was applied. Begg’s funnel plot asymmetry was explored for the presence of potential publication bias. In addition, the existence of heterogeneity was evaluated using I^2^ statistic and the Cochrane Q statistic. 

## Results

**Characteristics of the included studies**

In the present study, a total of 43 articles were collected from different databases and the reference lists. The selection process of the studies is illustrated in Figure 1. After screening studies based on the inclusion and exclusion criteria, 13 eligible articles were included in the meta-analysis. These studies had been conducted in Ahwaz, Hamadan, Karadj, Mashhad, Qazvin, Shahrekord, Shiraz, Tehran, and Tabriz in Iran (Table.1). As shown in the funnel plot, there was a possibility of publication bias in the eligible articles evaluating the prevalence of H. influenzae resistance to ampicillin. Similarly, the funnel plot was found to be asymmetric for many other antibiotics. Microbiological identification methods which were used for bacterial isolation from different specimens, such as cerebrospinal fluid, nasopharynx, and blood, were based on growth on chocolate agar medium at 37°C for 24-48 h in a candle jar, growth requirement for X (hemin) and V (NAD) factors, carbohydrate fermentation reactions, serological tests for serotyping, and biochemical tests for biotyping (e.g., oxidase, catalase, urea, indole, and ornithine decarboxylase).

**Characteristics of** **Haemophilus** **influenzae antibiotic resistance**

Most of the studies included in this review used Kirby-Bauer's disk diffusion method to determine the antimicrobial susceptibility of H. influenzae in Iran. In the present study, we observed a high degree of heterogeneity among the included studies when pooling data. Therefore, the prevalence of H. influenzae antibiotic resistance was evaluated using a random-effects model for most of the drugs. Haemophilus influenzae resistance rates to various antibiotics were as follows: 54.8% for ampicillin (95% CI: 40-68.7; I^2 ^=73.7%; Q=38; df (Q)=10; P=0.00), 66.6% for amoxicillin (95% CI: 43.6-83.7; I^2^=72.6%; Q=21.9; df (Q)=6; P=0.00), 28.6% for cephalexin (95% CI: 14.5-48.7; I^2^=0.0%; Q=1.6; df (Q)=2; P=0.43), 62% for cefixime (95% CI: 51.3-71.6; I^2^=25.2%; Q=5.3; df (Q)=4; P=0.25), 21.3% for ceftazidime (95% CI: 8.3-45; I^2^=75.9%; Q=8.3; df (Q)=2; P=0.01), 22.3% for cefotaxime (95% CI: 12.1-37.3; I^2^=0.0%; Q=1.7; df (Q)=2; P=0.41), 23.2% for ceftizoxime (95% CI: 16.4-31.8; I^2^=9%; Q=5.5; df (Q)=5; P=0.35), 27.7% for chloramphenicol (95% CI: 7.2-65.3; I^2^=73.8%; Q=22.9; df (Q)=6; P=0.00), 46.7% for tetracycline (95% CI: 17.8-78; I^2^=74.1%; Q=11.5; df (Q)=3; P=0.00), 53% for trimethoprim/sulfamethoxazole (95% CI: 36.1-69.3; I^2^=78.7%; Q=51.7; df(Q)=11; P=0.00), 82.6% for penicillin (95% CI: 75.2-88.2; I^2^=0.0%; Q=0.1; df (Q)=2; P=0.91), 40.3% for erythromycin (95% CI: 7.6-84.6; I^2^=74.1%; Q=7.7; df (Q)=2; P=0.02), 30.8% for ciprofloxacin (95% CI: 9.5-65.4; I^2^=68.4%; Q=9.4; df (Q)=3; P=0.02), 33.1% for ceftriaxone (95% CI: 19.5-50.4; I^2^=39.5%; Q=6.6; df (Q)=4; P=0.15), 40.2% for gentamicin (95% CI: 14.4-72.9; I^2^=74%; Q=19.2; df (Q)=5; P=0.00), 45.8% for amikacin (95% CI: 24.9-68.2; I^2^=51.6%; Q=6.2; df (Q)=3; P=0.10), 44.4% for kanamycin (95% CI: 7.3-89; I^2^=77.9%; Q=13.5; df (Q)=3; P=0.00), 18.5% for rifampin (95% CI: 7.4-39.3; I^2^=66.1%; Q=2.9; df (Q)=1; P=0.08), 17.4% for azithromycin (95% CI: 10.2-28.3; I^2^=0.0%; Q=0.9; df (Q)=1; P=0.34), and 71.3% for clindamycin (95% CI: 20.1-96.1; I^2^=84.6%; Q=13; df (Q)=2; P=0.00; Table.2). Additionally, other antibiotic resistance patterns were as follow: 6 (85%) for vancomycin, 7 (50%) for cefazolin, 6 (45.8%) for cephradine, 1 (25%) for ofloxacin, 0 (0%) for imipenem, 2 (100%) for cloxacillin, 9 (47.4%) for cephalothin, 16 (84.4%) for tobramycin, 6 (11.8%) for amoxicillin/clavulanic acid, 7 (13.7%) for cefuroxime, 18 (35.3%) for clarithromycin, and 3 (43%) for doxycycline.

## Discussion

According to the previous systematic reviews and meta-analysis, the most frequent causes of bacterial meningitis in Iran are Streptococcus pneumoniae, Hib, coagulase-negative staphylococci, and Neisseria meningitidis, respectively (23,24). In many countries, the introduction of conjugate Hib vaccines given during early infancy has led to reduced mortality rates, especially in children under 5 years of age living in developing countries (7,25). However, Hib vaccine is not compulsory in the vaccination program of Iran (25). Accordingly, bacterial meningitis is a health problem, especially in children in Iran, which requires antimicrobial treatment (12). The recommended empirical antibiotic therapy for bacterial meningitis in children and newborns is vancomycin plus cefotaxime or ceftriaxone (7,26). In the present study, the prevalence rates of vancomycin-, cefotaxime- and ceftriaxone-resistant H. influenzae strains in Iran were 85%, 22.3%, and 33.1%, respectively (Table.2). Additionally, chloramphenicol, cefepime, and meropenem are considered as alternative regimens (7,26). 

**Fig 2:**
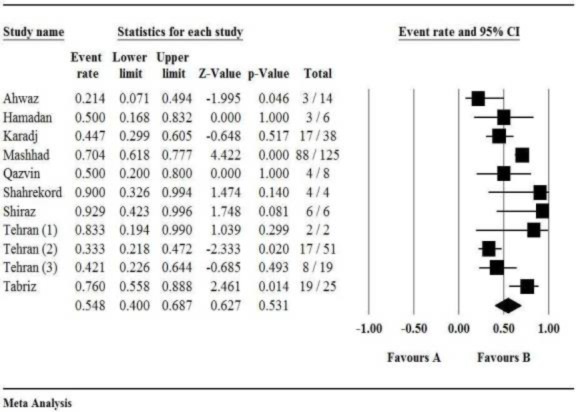
Forest plot of the meta-analysis of the prevalence of H. influenzae resistance to ampicillin in Iran

In Iran, the prevalence of chloramphenicol-resistant H. influenzae strains was 27.7%, and the rate of cefepime- and meropenem-resistant H. influenzae strains were not determined (Table.2). Furthermore, another alternative regimen is ampicillin plus cefepime or chloramphenicol for ampicillin-susceptible strains (7,26). The prevalence of H. influenzae strains resistant to ampicillin was estimated at 54.8% (Table.2 and Fig.2). 

**Fig 3:**
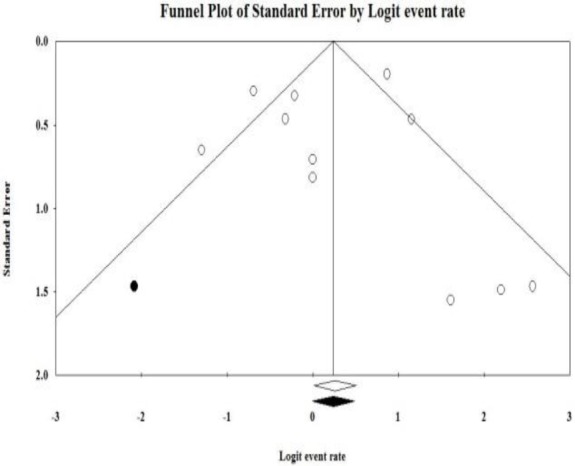
Funnel plot of the meta-analysis of the prevalence of H. influenzae resistance to ampicillin in Iran

**Table 1:** Profiles of the studies included in the meta-analysis

**Author (Ref)**	**Year**	**City**	**Strain (n)**	**AST**	**Antibiotic resistance (n)**																			
					**AMP**	**AMX**	**LEX**	**CFM**	**CAZ**	**CTX**	**ZOX**	**CHL**	**TET**	**TMP-SMX**	**PEN**	**ERY**	**CIP**	**CRO**	**GEN**	**AMK**	**KAN**	**RIF**	**AZM**	**CLI**
Farajzadeh (10)	2000-2001	Ahwaz	14	ND	3	4	5	ND	ND	2	3	1	2	8	ND	ND	ND	ND	ND	ND	ND	ND	ND	ND
Mashouf (11)	1998-2002	Hamadan	6	Disk diffusion	3	3	0	ND	ND	ND	0	0	ND	3	ND	ND	ND	ND	0	ND	0	ND	ND	ND
Mojgani (12)	ND	Karadj	38	ND	17	ND	ND	ND	ND	ND	ND	ND	15	18	ND	ND	ND	ND	ND	ND	ND	ND	ND	ND
Ghazvini (13)	2004-2005	Mashhad	125	Disk diffusion	88	99	ND	72	ND	ND	ND	ND	ND	89	103	22	ND	ND	ND	ND	ND	ND	ND	ND
Moafi (14)	2013-2014	Qazvin	8	Disk diffusion	4	4	2	ND	ND	ND	2	1	ND	4	ND	ND	ND	ND	1	ND	1	ND	ND	ND
Imani (15)	2005	Shahrekord	4	Broth micro dilution	4	ND	ND	ND	ND	ND	ND	ND	ND	ND	4	1	1	1	ND	ND	ND	ND	ND	ND
Shishegar (16)	2007-2008	Shiraz	6	Disk diffusion	6	6	ND	6	ND	0	ND	ND	ND	6	ND	6	4	0	ND	ND	ND	ND	ND	ND
Boroumand (17)	ND	Tehran	20	Disk diffusion	ND	19	ND	ND	ND	ND	ND	20	20	0	ND	ND	9	10	ND	ND	ND	ND	2	20
Kalantari(18)	1995-2005	Tehran	2	Disk diffusion	2	ND	ND	ND	ND	ND	ND	2	ND	2	2	ND	ND	ND	2	2	2	ND	ND	ND
Fahimzad (19)	2005	Tehran	51	Disk diffusion	17	ND	ND	30	5	ND	9	ND	ND	45	ND	ND	ND	ND	ND	ND	ND	6	10	15
Haghi (20)	2001-2007	Tehran	19	Disk diffusion	8	ND	ND	8	8	ND	8	7	ND	10	ND	ND	ND	8	12	10	16	ND	ND	ND
Dallal (21)	ND	Tehran	7	Disk diffusion	ND	4	ND	5	ND	ND	ND	ND	3	0	ND	ND	ND	ND	6	4	ND	ND	ND	5
Abdinia (22)	2003-2013	Tabriz	25	Disk diffusion	19	ND	ND	ND	5	7	5	0	ND	5	ND	ND	1	5	4	6	ND	7	ND	ND

**Abbreviations:** AMP: ampicillin, AMX: amoxicillin, LEX: cephalexin, CFM: cefixime, CAZ: ceftazidime, CTX: cefotaxime, ZOX: ceftizoxime, CHL: chloramphenicol, TET: tetracycline, TMP/SMX: trimethoprim/sulfamethoxazole, PEN: penicillin, ERY: erythromycin, CIP: ciprofloxacin, CRO: ceftriaxone, GEN: gentamicin, AMK: amikacin, KAN: kanamycin, RIF: rifampin, AZM: azithromycin, CLI: clindamycin, AST: antimicrobial susceptibility testing, ND: not determined, CSF: cerebrospinal fluid

**Table 2:** Antimicrobial susceptibility pattern of H. influenzae in Iran

**Province**	**Antibiotic resistance (%)(95% CI)**																			
	**AMP**	**AMX**	**LEX**	**CFM**	**CAZ**	**CTX**	**ZOX**	**CHL**	**TET**	**TMP-SMX**	**PEN**	**ERY**	**CIP**	**CRO**	**GEN**	**AMK**	**KAN**	**RIF**	**AZM**	**CLI**
Ahwaz	21.4 (7.1-49.4)	28.6 (11.1-56.1)	35.7 (15.7-62.4)	ND	ND	14.3 (3.6-42.7)	21.4 (7.1-49.4)	7.1 (1-37)	14.3 (3.6-42.7)	57.1 (31.6-79.4)	ND	ND	ND	ND	ND	ND	ND	ND	ND	ND
Hamadan	50 (16.8-83.2)	50 (16.8-83.2)	0	ND	ND	ND	0	0	ND	50 (16.8-83.2)	ND	ND	ND	ND	0	ND	0	ND	ND	ND
Karaj	44.7 (29.9-60.5)	ND	ND	ND	ND	ND	ND	ND	39.5 (25.4-55.6)	47.4 (32.3-63)	ND	ND	ND	ND	ND	ND	ND	ND	ND	ND
Mashhad	70.4 (61.8 -77.7)	79.2 (71.2-85.4)	ND	57.6 (48.8-66)	ND	ND	ND	ND	ND	71.2 (62.7-78.5)	82.4 (74.7-88.1)	17.6 (11.9-25.3)	ND	ND	ND	ND	ND	ND	ND	ND
Qazvin	50 (20-80)	50 (20-80)	25 (6.3-62.3)	ND	ND	ND	25 (6.3-62.3)	12.5 (1.7-53.7)	ND	50 (20-80)	ND	ND	ND	ND	12.5 (1.7-53.7)	ND	12.5 (1.7-53.7)	ND	ND	ND
Shahrekord	90 (32.6-99.4)	ND	ND	ND	ND	ND	ND	ND	ND	ND	90 (32.6-99.4)	25 (3.4-76.2)	25 (3.4-76.2)	25 (3.4-76.2)	ND	ND	ND	ND	ND	ND
Shiraz	92.9 (42.3-99.6)	92.9 (42.3-99.6)	ND	92.9 (42.3-99.6)	ND	0	ND	ND	ND	92.9 (42.3-99.6)	ND	92.9 (42.3-99.6)	66.7 (26.8-91.6)	0	ND	ND	ND	ND	ND	ND
Tehran	37.1 (26.6-48.9)	82.2 (25.5-98.4)	ND	55.7 (44.4-66.5)	22 (4.2-64.5)	ND	27.6 (10.4-55.8)	79.4 (19.8-98.4)	82.2 (8.5-99.6)	43.5 (11-82.7)	83.3 (19.4-99)	ND	45 (25.3-66.4)	46.2 (31.3-61.7)	69.1 (49.6-83.6)	56 (37.6-72.9)	84.1 (62.8-94.3)	11.8 (5.4-23.8)	17.4 (10.2-28.3)	71.3 (20.1-96.1)
Tabriz	76 (55.8-88.8)	ND	ND	ND	20 (8.6-40)	28 (14-48.2)	20 (8.6-40)	0	ND	20 (8.6-40)	ND	ND	4 (0.6-23.5)	20 (8.6-40)	16 (6.1-35.7)	24 (11.2-44.2)	ND	28 (14-48.2)	ND	ND
Total	54.8 (40-68.7)	66.6% (43.6-83.7)	28.6 (14.5-48.7)	62 (51.3-71.6)	21.3 (8.3-45)	22.3 (12.1-37.3)	23.2 (16.4-31.8)	27.7 (7.2-65.3)	46.7 (17.8-78)	53 (36.1-69.3)	82.6 (75.2-88.2)	40.3 (7.6-84.6)	30.8 (9.5-65.4)	33.1 (19.5-50.4)	40.2 (14.4-72.9)	45.8 (24.9-68.2)	44.4 (7.3-89)	18.5 (7.4-39.3)	17.4 (10.2-28.3)	71.3 (20.1-96.1)

AMP: ampicillin, AMX: amoxicillin, LEX: cephalexin, CFM: cefixime, CAZ: ceftazidime, CTX: cefotaxime, ZOX: ceftizoxime, CHL: chloramphenicol, TET: tetracycline, TMP/SMX: trimethoprim/sulfamethoxazole, PEN: penicillin, ERY: erythromycin, CIP: ciprofloxacin, CRO: ceftriaxone, GEN: gentamicin, AMK: amikacin, KAN: kanamycin, RIF: rifampin, AZM: azithromycin, CLI: clindamycin, ND: not determined

Fluoroquinolone antibiotics are also recommended for adult patients (7,26). According to our results, 30.8% and 25% of H. influenzae strains were resistant to ciprofloxacin and ofloxacin, respectively.

After meningitis, childhood pneumonia and bacteremia are the most common diseases caused by Hib strains, and pneumonia is particularly dominant in developing countries (27). Amoxicillin or amoxicillin/clavulanic acid (co-amoxiclav) are recommended for outpatients. Furthermore, ceftriaxone or cefotaxime are suggested for inpatients with pediatric pneumonia empirical therapies (7,28). Ceftriaxone, cefotaxime, or cefuroxime are also suggested for the treatment of pneumonia and bacteremia caused by β-lactamase-producing H. influenzae strains. On the other hand, ampicillin is suggested for β-lactamase-negative strains (7,28). The prevalence rates of H. influenzae strains resistant to amoxicillin, amoxicillin/ clavulanic acid, and cefuroxime in Iran were 66.6%, 11.8%, and 13.7%, respectively.

Untypeable H. influenzae is responsible for 2-12% of community-acquired pneumonia. The recommended antibiotics for this strain are azithromycin, clarithromycin, and doxycycline (7). Azithromycin and clarithromycin are also alternative treatments in patients with acute otitis media who have penicillin allergy. Based on the evidence, 23-67% of acute otitis media cases are caused by untypeable H. influenzae (7,29). In Iran, 17.4%, 35.3%, and 43% of H. influenzae strains were reported to be resistant to azithromycin, clarithromycin, and doxycycline, respectively. Trimethoprim-sulfamethoxazole, erythromycin, rifampin, and cefixime, along with many other antibiotics, were used to treat acute sinusitis caused by untypeable H. influenzae in adult and pediatric patients (7). In the present study, the prevalence rates of H. influenzae strains resistant to trimethoprim-sulfamethoxazole, erythromycin, rifampin, and cefixime were 53%, 40.3%, 18.5%, and 62%, respectively. 

Our results were compared with those of other studies performed in other countries. In this regard, H. influenzae antibiotic resistance rate to ampicillin in Iran (54.8%) was found to be higher than those reported for Lebanon (17.4%), France (43%), Germany (20.1%), Italy (11.4%), Mexico (27.4%), and South Africa, Spain, and United States (0%) (5,30). Furthermore, the antibiotic resistance rates of H. influenzae to amoxicillin-clavulanate (11.8%), azithromycin (17.4%), and ceftriaxone (33.1%) were higher in comparison to those reported in Turkey (azithromycin: 0%), Korea (amoxicillin-clavulanate: 10.4%), and France, Germany, Italy, Mexico, South Africa, Spain, and United States (0%) (5,31,32). 

Clarithromycin (35.3%), chloramphenicol (27.7%), trimethoprim/sulfamethoxazole (53%), and cefotaxime (22.3%) had higher resistance rates in Iran than in Lebanon, Korea, Turkey, North America, Latin America, and Europe (5,30-32). Similarly, antibiotic resistance rates to quinolones, ciprofloxacin (30.8%), and ofloxacin (25%) were higher than those reported in Lebanon, North America, Latin America, and Europe (5,30). The H. influenzae antibiotic resistance rates to rifampin (18.5%) and tetracycline (46.7%) were higher as compared to those in Lebanon and Spain (30,33). Finally, antibiotic resistance rates to gentamicin, amikacin, and kanamycin were higher than that reported for Lebanon (30), while resistance to ampicillin and cefuroxime was lower than that of Korea (32).

The initial origin of many antibiotic-resistant strains, such as sulfonamide-resistant Streptoccoccus pyogenes, penicillin-resistant Staphylococcus aureus, and streptomycin-resistant Mycobacterium tuberculosis, were hospitals, where drugs are used extensively (34). Such bacteria, along with the emergence of multidrug-resistant strains, have become a health concern and led to treatment failure and increased healthcare costs, especially in developing countries where antibiotics are available without a prescription (34). Therefore, it is essential to manage and prevent drug resistance by tracking drug resistance rates at national and international levels. Isolation of individuals in hospitals who are infected with difficult-to-treat bacterial agents is another measure to prevent the spread of resistant strains in hospitals and communities. Finally, the provision of rapid diagnostic and new therapeutic methods (e.g., reduced or careful use of current antibiotics and development of new antimicrobials and vaccines) are necessary to control the spread of antibiotic-resistant strains (34).

## Conclusion

The results of the reviewed studies were indicative of an alarming trend in H. influenzae resistance to the majority of antibiotic drugs tested in Iran. Continuation of this trend will reduce therapeutic options and complicate the successful management of H. influenzae infections. Therefore, several precautionary measures are essential to be implemented. Some of these measures include: 1) creating a strong surveillance system in order to allow continuous monitoring of drug resistance, 2) enhancing physicians’ awareness about drug resistance trends and avoiding prescribing antibiotics with a high resistance rate, 3) informing patients about drug resistance trends in order to avoid misusing antibiotics to prevent the prevalence of antibiotic-resistant pathogens, 4) further investigating the resistance mechanisms of H. influenzae against different antimicrobial agents in Iran, and 5) performing continuous antimicrobial susceptibility tests to select the most effective drugs or use new antibiotics. 
